# Robust Heart Sound Analysis With MFCC and Light Weight Convolutional Neural Network

**DOI:** 10.1109/OJEMB.2025.3615395

**Published:** 2025-09-29

**Authors:** Aliya Hasan, Mohammad Karim

**Affiliations:** Department of Electrical and Computer EngineeringUniversity of Massachusetts Dartmouth 14709 Dartmouth MA 02747 USA

**Keywords:** Auscultation, convolutional neural network (CNN), deep learning, heart disease, mel-frequency cepstral coefficients

## Abstract

*Objective:* Heart sound analysis is essential for cardiovascular disorder classification. Traditional auscultation and rule-based methods require manual feature engineering and clinical expertise. This work proposes a CNN-based model for automated multiclass heart sound classification. *Results:* Using MFCC features extracted from segmented real-world recordings, the model classifies heart sounds into murmur, extrasystole, extrahls, artifact, and normal. It achieves 98.7% training accuracy and 91% validation accuracy, with strong precision and recall for normal and murmur classes, and a weighted F1-score of 0.91. *Conclusions:* The results show that the proposed MFCC-CNN framework is robust, generalizable, and suitable for automated auscultation and early cardiac screening.

## I. Introduction

Cardiovascular diseases (CVDs) are the leading cause of death worldwide, responsible for approximately 17.9 million deaths annually—32% of global mortality, as per the WHO [1]. Many of these deaths result from delayed diagnosis or limited access to screening. Auscultation, a non-invasive and widely used method for detecting heart anomalies, depends heavily on clinician expertise, introducing subjectivity and limiting consistency.

While early detection can significantly improve outcomes, advanced diagnostics such as echocardiograms and electrocardiograms (ECGs) are resource-intensive and often unavailable in underserved areas. This continues to drive the development of automated heart sound classification systems to support clinical decision-making.

Heart sound analysis has played a vital role in the early diagnosis of cardiovascular diseases (CVDs). Over the past two decades, numerous studies have explored the automation of auscultation using digital signal processing and machine learning techniques. Traditional methods employed wavelet transforms, short-time Fourier transforms (STFT), and cepstral analysis to extract time-frequency features, which were classified using algorithms such as Support Vector Machines (SVM), k-Nearest Neighbors (k-NN), and decision trees. Springer et al. [2] introduced a segmentation method using hidden Markov models (HMMs) to detect the first (S1) and second (S2) heart sounds in phonocardiograms (PCGs), which enhanced classification reliability but required precise segmentation and domain knowledge.

Potes et al. [3] used an ensemble of convolution neural networks (CNNs) and recurrent neural networks (RNNs) to classify heart sounds as normal or abnormal, performing well in the PhysioNet/CinC Challenge but limited to binary classification. Zhang et al. [4] applied deep residual networks with mel-spectrograms for murmur detection. Both approaches focused on two or three classes, overlooking detailed categories such as artifacts and extrahls.

Recent work applied end-to-end CNNs with mel-frequency cepstral coefficients (MFCCs), improving accuracy and efficiency without manual features. Noman et al. [5] achieved 89.22% accuracy on the Pascal dataset using CNNs with MFCCs. Yaseen et al. [6] combined MFCCs with discrete wavelet transform (DWT) and classifiers such as SVM, DNNs, and k-NN, improving accuracy. Syed et al. [7] used cepstral coefficients and spectral entropy with artificial neural networks (ANNs) but showed limited generalization. Choi and Jiang [8] applied energy-based segmentation and principal component analysis (PCA) with decision trees, while Mandal et al. [9] used wavelet decomposition with SVM for murmur detection. These methods were effective but noise-sensitive and dependent on feature selection.

Deep learning has revolutionized biomedical signal classification by learning hierarchical features. CNNs, in particular, are effective in capturing patterns from spectrograms and MFCCs. Gharehbaghi and Lindén [10] used stacked CNNs on time-frequency images of PCG signals and achieved high accuracy but at high computational cost. Tschannen et al. [11] introduced data augmentation techniques to improve CNN performance. Cheng et al. [12] combined CNNs and attention mechanisms for murmur region detection in long PCG recordings, though these models required large datasets and training time.

Rubin et al. [13] converted audio features into images for deep CNNs, achieving strong performance. Chowdhury et al. [14] used spectrograms with CNNs for binary classification and achieved 89.2% accuracy. Nogueira et al. [15] combined MFCCs with SVM and Random Forest (RF) classifiers, attaining 83.22% accuracy. Khan et al. [16] used MFCCs and Long Short-Term Memory (LSTM) networks with log-Mel spectrograms, reaching 95.75% accuracy. Yang and Hsieh [17] applied RNNs to detect anomalies, reaching 80% accuracy. Humayun et al. [18] developed a learnable filter bank CNN with time-convolutional (tConv) layers, achieving 81% accuracy and robustness to sensor variability. Yuvaraj et al. [19] and Chen et al. [20] also used stacked CNNs and attention mechanisms, but required high computational demands.

Bhatt et al. [21] proposed an ensemble method using DenseNet and SVM for multiclass PCG classification, achieving a 91.4% F1-score. Lightweight CNNs like SqueezeNet and MobileNet have emerged to support real-time deployment [22]. Hassanuazzaman et al. [23] proposed a transformer-based CNN for classifying short-segment pediatric heart sounds, demonstrating the value of attention mechanisms in short-duration recordings. Despite progress, most existing models are limited to binary or ternary classification and show reduced performance when generalizing across datasets or handling complex, noise-sensitive classes like extrahls and artifacts.

Dhar et al. [24] proposed a hybrid model using ensemble learning (e.g., RF, stacking classifiers) and deep learning (ANN, LSTM, BiLSTM) for binary and multiclass classification across datasets. Their system achieved 94.60% for binary and 77.77% for multiclass classification and included a Streamlit-based web app for real-time use.

Recent studies [25], [26] explored CNN-based diagnostic systems in clinical settings, underscoring the growing role of audio signals in healthcare. In contrast, our model focuses on fine-grained heart sound classification with greater clinical specificity.

This research proposes a deep learning-based approach using a convolutional neural network (CNN) trained on Mel-Frequency Cepstral Coefficients (MFCCs) extracted from heart sound recordings. MFCCs effectively capture key frequency and temporal patterns, useful for identifying cardiac cycle sounds like the "lub" and "dub." Unlike traditional systems based on k-Nearest Neighbors (k-NN), Support Vector Machines (SVM), or decision trees, the proposed CNN autonomously learns complex features from data, enhancing accuracy and robustness in multiclass tasks—Normal, Murmur, Extrasystole, Extrahls, and Artifact.

This study investigates whether a lightweight CNN trained on MFCC features can accurately classify five heart sound types and generalize effectively across diverse recording conditions.

The paper is organized as follows: Section II presents results, Section III discusses findings, Section IV gives the conclusion, and Section V outlines materials and methods.

Table 1 summarizes our scalable, lightweight CNN using MFCC features from 3-second PCG segments. The model eliminates manual segmentation, learns directly from raw audio, and classifies five heart sound types. Trained on multiple public datasets, it generalizes across recording conditions and accurately distinguishes Normal, Murmur, Extrasystole, Extrahls, and Artifact while remaining computationally efficient.

**TABLE 1:** Key Architectural Differences Between Existing and Proposed Models

Feature	Binary or Ternary Model (Existing)	Proposed Model
**Input Length / Segment**	Often 5-10 sec, not standardized	Fixed 3-sec slices to reduce variability
**Feature Extraction**	Simple MFCCs or Wavelets	MFCC + Delta + possibly log-Mel or multi-resolution
**Model Depth**	Shallow CNN or hybrid	Optimized CNN with GAP & Dropout for regularization
**Output Classes**	Normal, Murmur, Extrasystole (typically)	Normal, Murmur, Extrasystole, Artifact, Extrahls
**Dataset Handling**	Often imbalanced, low diversity	Pre-processed, balanced multi-class samples
**Generalization Strategy**	Rare	Better generalization with dropout and early stopping

## II. Results

The proposed CNN model was evaluated on a curated dataset with five classes: Normal, Artifact, Murmur, Extrasystole, and Extrahls. Using 3-second windowing, MFCCs were extracted as 2D feature maps and input to the CNN for training and classification.

### A. Performance Metrics

To ensure fair evaluation across all classes, we used macro and weighted averages along with confusion matrices.

**Figure 1.**
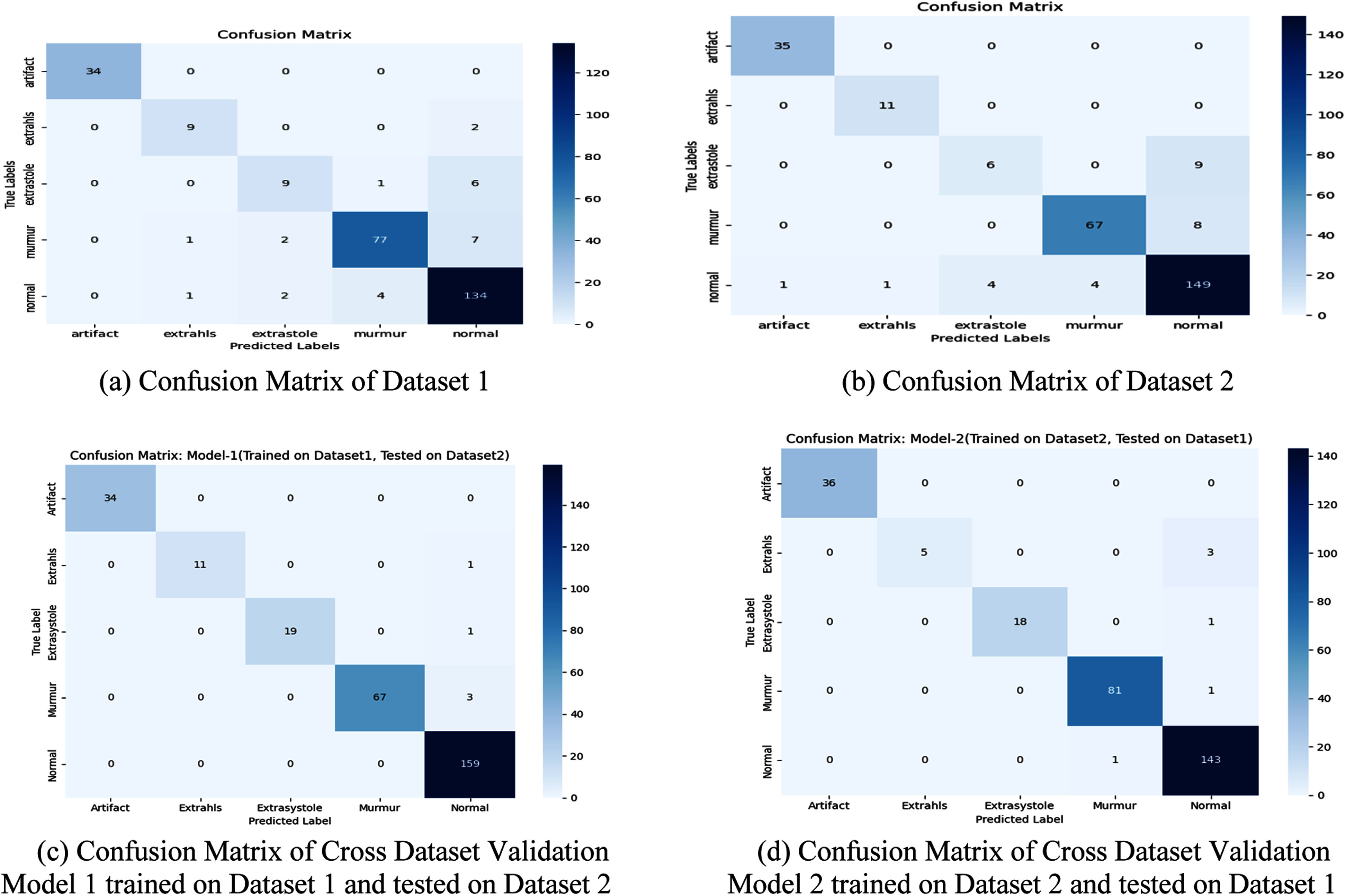
(a) Confusion matrix of dataset 1. (b) Confusion matrix of dataset 2. (c) Confusion matrix of cross dataset validation. (d) Confusion matrix of cross dataset validation model 1 trained on dataset 1 and tested on dataset 2 model 2 trained on dataset 2 and tested on dataset 1.

To evaluate model performance, standard metrics accuracy, precision, recall, and F1-score [27]—were applied using (1) to (7), along with confusion *matrices* for Dataset 1 and Dataset 2 shown in Fig. 1(a) and (b).

\begin{align*}
\text{Precision} =& \frac{{TP\ }}{{TP + FP}}\ \tag{1}\\
{\mathrm{Recall\ }} =& \frac{{TP\ }}{{TP + FN}} \tag{2}\\
F1 - \text{Score} =& 2{\mathrm{\ X\ }}\frac{{{\mathrm{Precision\ X\ Recall}}}}{{\text{Precision} + \text{Recall}}} \tag{3}\\
{\mathrm{Accuracy }} =& \frac{{TP + TN}}{{TP + TN + FP + FN}}\ \tag{4}\\
{\mathrm{Macro Average }} =& \frac{1}{N}\ \ \mathop \sum \limits_{i = 1}^N \text{Metric}{{\ }_{i\ }} \tag{5}\\
{\mathrm{Weighted\ Average }} =& \ \mathop \sum \limits_{i = 1}^N {{\mathrm{w}}_{i\ \ }}.{\mathrm{\ Metric}}{{\ }_{i\ }} \tag{6}\\
\text{where}\,{\mathrm{ }}{{\mathrm{w}}_{i\ \ }} =& \ \frac{{{\mathrm{Support\ }}{{\ }_{i\ }}}}{{Total\ \text{Support}}} \tag{7}
\end{align*}

Here, TP, TN, FP, and FN denote True Positives, True Negatives, False Positives, and False Negatives. *N* is the number of classes, and Support*i* is the count of true instances for class i. Fig. 1(c) and (d) respectively show confusion matrices of cross dataset validation for (i) Model 1 trained on Dataset 1 and tested on Dataset 2; and (ii) Model 2 trained on Dataset 2 and tested on Dataset 1.

**Figure 2.**
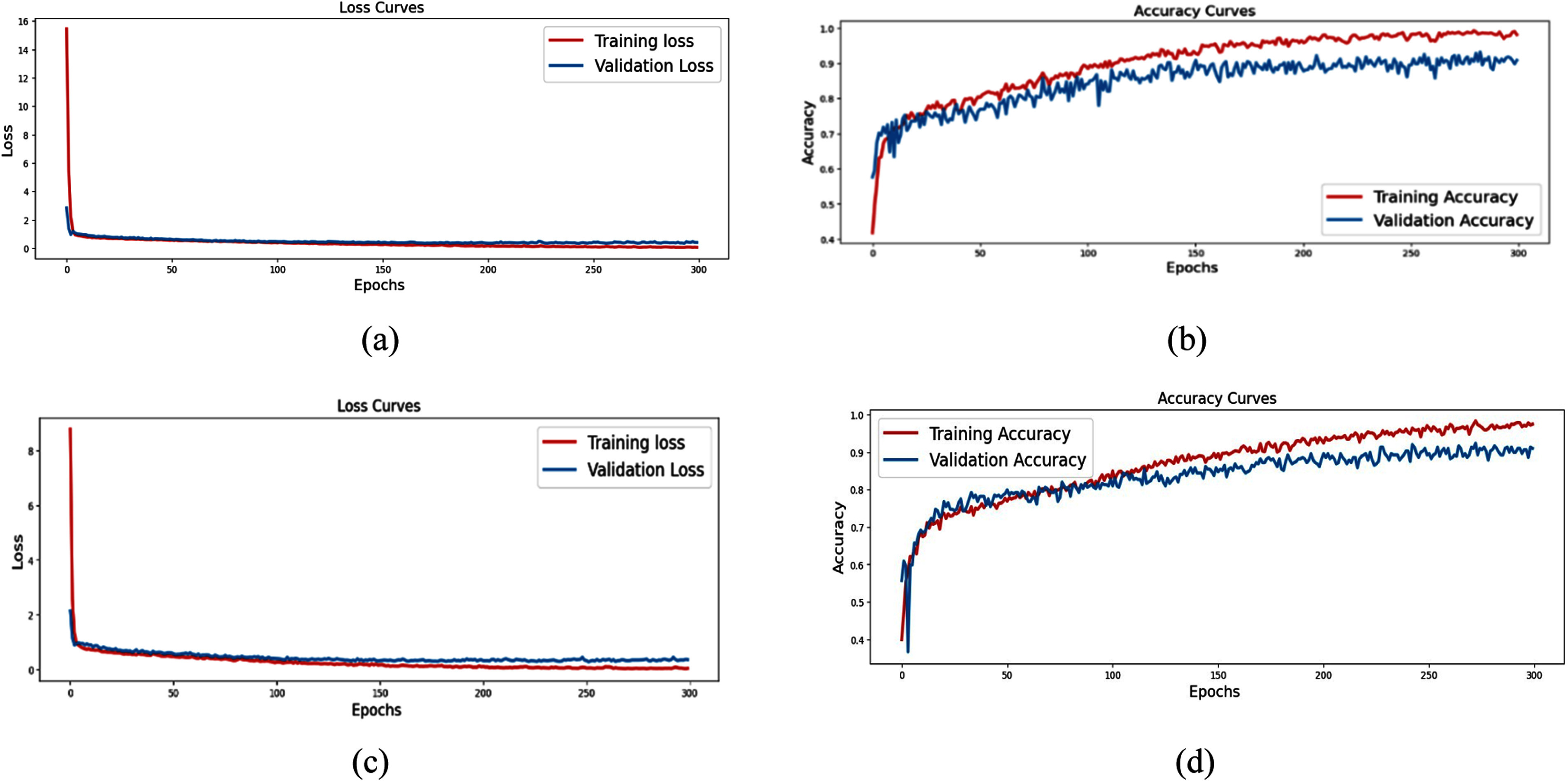
(a) Loss curve for dataset 1. (b) Accuracy curve for dataset 1. (c) Loss curve for dataset 2. (d) Accuracy curve for dataset 2.

The model achieved 91% validation accuracy on both datasets, with training accuracies of 98.7% on Dataset 1 and 99.85% on Dataset 2—demonstrating reliable classification across all five classes. The confusion matrices show minimal misclassifications. Notably, the model effectively distinguishes between similar-sounding classes like Murmur and Extrahls, which are often confused by clinicians and traditional algorithms. Fig. 2(a)–(d) shows the accuracy and loss curves of proposed model. Training accuracy surpasses validation accuracy over a wider epoch range in Dataset 2 than in Dataset 1. This observation may indicate slight overfitting in Dataset 2, likely due to its smaller number of samples (585 vs 832 in Dataset 1). Additionally Dataset 1 includes recordings from more diverse sources, which may have helped the model converge faster and generalize better. These training dynamics are reflected in the accuracy and loss curves shown in Fig. 2.

The confusion matrices in Fig. 1 show that most misclassifications occur between Extrasystole and Extrahls, and between Murmur and Extrahls. These errors are understandable due to overlapping acoustic patterns, such as S3 and S4 components, which can resemble murmurs—especially in noisy environments. This suggests the need for more refined features or enhanced denoising to reduce ambiguity.

### B. Class-Wise Performance

Table 2 shows class-wise results for both datasets. Normal and Artifact classes achieved the highest accuracy due to distinct signals. Murmur and Extrasystole saw minor misclassification from acoustic similarity, yet all classes maintained precision and recall above 97%. The Extrahls class, containing additional heart sounds like S3 and S4, was also accurately identified, confirming the model's multiclass effectiveness.

**TABLE 2:** Classification Performance Metrics For Heart Sound Classes

	Dataset 1				Dataset 2			
Class	**Precision**	**Recall**	**F1 Score**	**Support**	**Precision**	**Recall**	**F1-Score**	**Support**
Artifact	1	1	1	34	1	0.97	0.99	36
Extrahls	0.82	0.82	0.82	11	1	0.92	0.96	12
Extrasystole	0.56	0.69	0.62	13	0.4	0.6	0.48	10
Murmur	0.89	0.94	0.91	82	0.89	0.94	0.92	71
Normal	0.95	0.9	0.92	149	0.94	0.9	0.92	166
**Accuracy**			**0.91**	**289**			**0.91**	**295**
**Macro Avg**	0.84	0.87	0.85	289	0.85	0.87	0.85	295
**Weighted Avg**	0.92	0.91	0.91	289	0.92	0.91	0.91	295

To address data variability, this study used two public datasets—training on one and validating on the other—to evaluate generalization. The proposed 5-class CNN model consistently achieved 91% accuracy on both. Artifact and Murmur showed strong performance with F1-scores above 0.90. However, Extrasystole (recall: 0.69, 0.60) and Extrahls (recall: 0.82, 0.92) had lower recall, likely due to class imbalance and acoustic similarity. Still, macro and weighted F1 scores remained balanced (both 0.85), confirming robustness and potential for real-world use.

### C. Cross-Dataset Training and Testing

To evaluate the generalizability and robustness of the proposed model, cross-dataset training and testing were conducted using two independent heartbeat sound datasets. Model 1 was trained on Dataset 1 and evaluated on Dataset 2, while Model 2 was trained on Dataset 2 and tested on Dataset 1. This cross-domain evaluation simulates real-world deployment scenarios where the model may encounter unseen data distributions. Confusion matrices shown in Fig. 1(c) and (d) and classification reports shown in Table 3 confirms the model's capability to accurately distinguish between five heart sound classes, even when tested on a dataset with different recording conditions and sources. Cross-dataset evaluation confirmed strong generalization. Both models achieved 98% accuracy, indicating robustness to data variability.

• *Class-wise:* Artifact, Murmur, and Normal had high precision and recall. Extrasystole showed lower recall in Model 2 (0.62), likely due to class imbalance or inter-dataset variation.
• *Averages:* Macro F1 dropped in Model 2 (0.94 vs. 0.98), though weighted F1 score remained at 0.98.
• *Implication:* Results validate the model's robustness, though better representation of minor classes may improve cross-domain performance.

**TABLE 3:** Classification Performance Metrics Cross-Dataset Testing

	Model -1 (Trained on Dataset1, Tested on Dataset2)				Model -2 (Trained on Dataset2, Tested on Dataset1)			
Class	**Precision**	**Recall**	**F1 Score**	**Support**	**Precision**	**Recall**	**F1-Score**	**Support**
Artifact	1	1	1	34	1	1	1	36
Extrahls	1	0.92	0.96	12	1	0.62	0.77	8
Extrasystole	1	0.95	0.97	20	1	0.95	0.97	19
Murmur	1	0.96	0.98	70	0.99	0.99	0.99	82
Normal	0.97	1	0.98	159	0.97	0.99	0.98	144
**Accuracy**			0.98	295			0.98	289
**Macro Avg**	0.99	0.96	0.98	295	0.99	0.91	0.94	289
**Weighted Avg**	0.98	0.98	0.98	295	0.98	0.98	0.98	289

### D. Comparative Evaluation

Compared to prior models, the proposed method, as shown in Table 4, offers superior accuracy and broader class coverage. Unlike earlier binary or ternary models, it effectively handles multi-class classification without explicit segmentation or handcrafted features, simplifying pre-processing.

**TABLE 4:** Proposed Method Versus Those in Existing Literature

Author & References	Features Used	Classifier/Model	No. of Classes	Accuracy (%)	Remarks
Springer et al. [2]	Time-domain, Logistic-HSMM	Logistic Regression, HMM	Binary	∼86	Relies on segmentation, limited classes
Potes et al. [3]	MFCC + handcrafted	CNN + RNN Ensemble	Binary	∼87	High performance, but limited class scope
Noman et al. [5]	MFCC	Ensemble CNN	Ternary	89.22	Basic CNN, not optimized for large dataset
Cheng et al. [6]	MFCC + Spectrogram	2D-CNN	Ternary	∼92	Complex architecture, high training time
Chowdhury et al. [14]	Spectrograms	CNN-based spectrogram classification	Binary	∼89	Demonstrated CNN's capability in capturing local time-frequency features
Nogueira et al. [15]	MFCC features	SVM and RF	Binary	83.22	Robust to noise
Khan et al [16]	MFCC	LSTM	Binary	95.75	Good performance, high memory footprint
Yang and Hsieh [17]	1D time- series signals	RNN	Binary	∼80	Requires large training data
Humayun et al. [18]	1D time- series signals	Conv-CNN (1D-CNN)	Binary	∼81	Good performance, high memory footprint
**Proposed Work**	MFCC (from 3s audio window)	Customized CNN	**Dataset 1:** 98.7 –Training 91 – Validation **Dataset 2 :** Five Class 99.85 – Training 91 – Validation		Lightweight, robust to noise, multiclass

## III. Discussion

The proposed CNN performed strongly across five heart sound categories. Normal and Artifact had the highest accuracy, while Murmur and Extrasystole showed overlap with Extrahls, reflecting the acoustic similarity of S3 and S4 to murmurs.

Confusion matrices showed misclassifications mainly among these classes. This limitation is common in practice and suggests refinements such as denoising or additional feature extraction could improve separability.

Cross-dataset evaluation showed robustness across recording conditions, though recall for Extrasystole declined due to imbalance and variability. Still, high weighted F1-scores confirmed good generalization to unseen data.

Unlike earlier binary or ternary approaches, this framework distinguishes detailed classes such as artifacts and extrahls, supporting integration into diagnostic systems. Future work will expand datasets, explore features *like* wavelets or raw waveforms, and validate performance on diverse devices and noisy environments, with clinical collaborations planned to assess real-world utility.

## IV. Conclusion

This study presented a lightweight CNN framework using MFCC features for five-class heart sound classification. The model achieved 98.7% training accuracy and 91% validation accuracy, outperforming prior binary and ternary methods while maintaining generalization across datasets. Its ability to handle acoustically similar classes and its computational efficiency make it suitable for real-time diagnostic use, including in resource-limited settings.

Future work will emphasize clinical validation and deployment, supporting the transition of this approach from research to practical healthcare applications in mobile and point-of-care diagnostic systems.

## V. Materials and Methods

**Figure 3.**
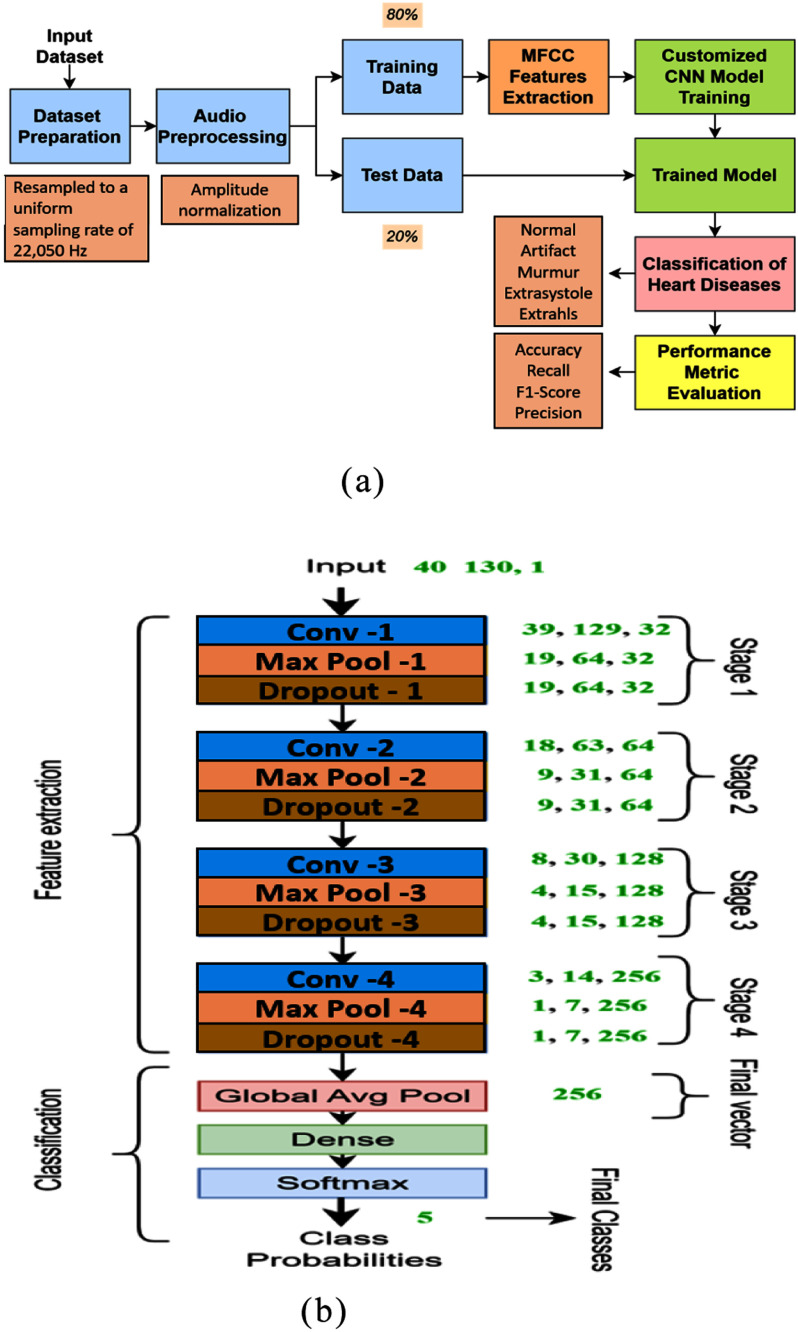
(a) Proposed model for heart sound classification. (b) Architecture of the proposed CNN model.

This research presents a CNN-based deep learning approach for heart sound classification using MFCCs. The overall methodology, illustrated in Fig. 3(a), includes dataset preparation, audio preprocessing and segmentation, MFCC feature extraction, CNN model training, and performance evaluation—each described in detail below. Fig. 3(b) presents the architecture of the proposed CNN model, outlining the convolution, pooling, and dropout layers followed by the classification stage. Conv-1 through Conv-4 correspond to the four convolutional layers in the proposed CNN, with increasing filter depths of 32, 64, 128, and 256. Each layer is followed by max-pooling and dropout, enabling progressive extraction of higher-level MFCC features for classification.

### A. Dataset Preparation

The datasets used in this research comprise labeled heart sound recordings sourced from publicly available repositories on Kaggle [28]. The recordings span five heart sound classes: normal, murmur, extrasystole, extrahls, and artifact. The Heart Disease dataset **(**Dataset 1) includes Set A, collected via an iPhone app from the general public, and Set B, recorded in clinical settings using digital stethoscopes. Metadata and labels are provided in set_a.csv and set_b.csv, with set_a_timing.csv offering gold-standard annotations for Set A. Audio durations range from 1 to 30 seconds, with noise-prone segments trimmed.

The Heartbeat Sound dataset (Dataset 2) consists of 832 PCG recordings across six folders: normal (351), murmur (129), extrasystole (46), extrahls (19), artifact (40), and unlabel (247). For supervised learning, the unlabel category was excluded, yielding 585 labeled samples. This 5-class curated dataset captures a wide range of cardiac conditions used to train and evaluate the proposed model.

**Figure 4.**
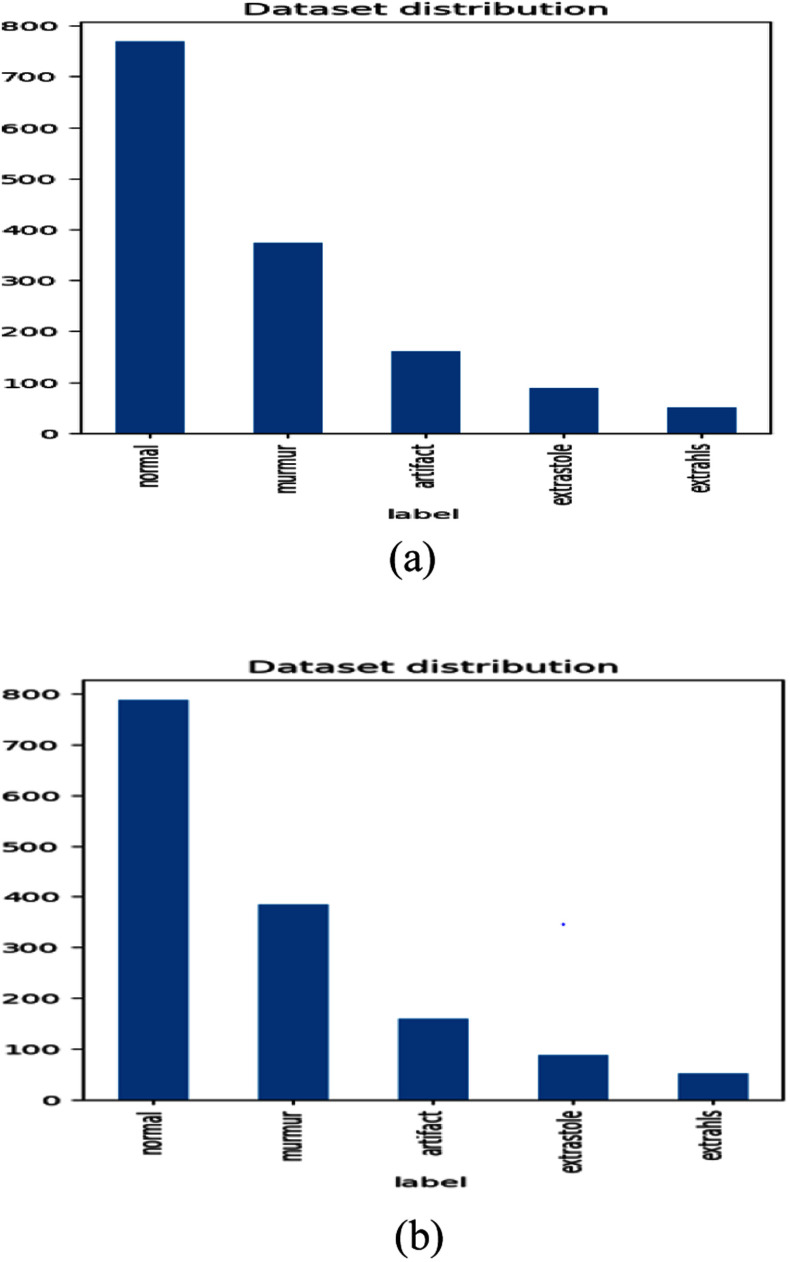
Dataset distributions: (a) Dataset 1 distribution, (b) dataset 2 distribution.

All recordings are resampled to 22050 Hz to keep the data consistent. Each audio file is split into overlapping 3-second segments to capture full heart cycles and increase training samples. Using fixed-length clips helps keep the input size uniform. Fig. 4 shows the distribution of the two datasets.

### B. Audio Preprocessing

Each audio segment was preprocessed for quality and consistency. Amplitude normalization equalized loudness, and spectral gating reduced noise when interference was observed. Overlapping windows with 50% overlap (1.5 s offset) allowed longer recordings to contribute more data while preserving continuity.

A fixed 3-second window was selected as it typically captures at least two full heart cycles, balancing signal completeness with consistent input shape. Preliminary tests with 2s and 4s segments showed that 3s offered the best model performance.

### C. Feature Extraction

Using MFCC the preprocessed audio clips are transformed into feature representations commonly used in sound and speech recognition. A mel-spectrogram is computed with 128 mel bands, a frame size of 2048, and a hop length of 512. This spectrogram is converted to a decibel scale to extract 40 MFCCs per frame. These coefficients capture both spectral and temporal characteristics of heart sounds and are arranged as 2D matrices, which serve as grayscale image inputs to the CNN.

A configuration of 40 MFCCs, a frame size of 2048, and a hop length of 512 was chosen based on prior studies and preliminary testing, as it consistently yielded stable and accurate classification results.

### D. CNN-Based Classification Model

An overview of the proposed CNN architecture for heart sound classification is illustrated in Fig. 3(b), detailing the input processing, convolutional layers, and output classification flow.

A custom CNN is designed to classify the MFCC features extracted from heart sound recordings. The model starts with a 2D convolutional layer using 32 filters to capture low-level features, followed by max pooling to reduce spatial dimensions and improve efficiency. A dropout layer is applied next to prevent overfitting. This pattern repeats with deeper convolutional layers using 64, 128, and 256 filters, each followed by pooling and dropout layers. These layers progressively learn more abstract patterns from the input data.

After the final convolutional block, a Global Average Pooling (GAP) layer compresses each feature map into a single scalar, reducing parameters and improving generalization. The final dense layer has five neurons representing the classes—artifact, extrahls, extrasystole, murmur, and normal—using softmax activation for multi-class classification. The architecture includes approximately 173925 trainable parameters, making it lightweight and suitable for real-time deployment.

This CNN balances depth, regularization, and efficiency, effectively learning complex heart sound patterns and achieving strong classification performance.

The design choices—such as using 3-second MFCC slices, GAP for dimensionality reduction, and dropout for regularization—were motivated by the need to create a lightweight model that generalizes well across varying datasets. This balance of complexity and efficiency allows the model to effectively capture temporal-spectral characteristics while being deployable on edge devices.

The model was trained using the Adam optimizer (learning rate = 0.001), batch size of 32, and categorical cross-entropy loss for 50 epochs. The CNN includes four convolutional layers with 3×3 kernels, ReLU activation, and 2×2 max pooling. Dropout (rate = 0.3) follows each block. A GAP layer and softmax-activated dense output layer complete the architecture.

### E. Key Architectural Differences Between Existing and Proposed Models

Table 1 highlights key differences between existing and proposed models. Traditional methods typically use a CNN + SVM pipeline with MFCC or spectrogram features for up to three classes—Normal, Murmur, and Extrasystole. They often require handcrafted preprocessing and segmentation, limiting scalability and adaptability.

The proposed model features a lightweight custom CNN with dropout and GAP layers, operating on MFCCs from 3-second PCG segments. It supports end-to-end learning, removes the need for manual segmentation, and accurately classifies five clinically relevant classes, showing strong generalizability across datasets.
